# Relationships between dyadic coping, marital adjustment, and post-traumatic growth in patients with maintenance hemodialysis patients and their spouses

**DOI:** 10.3389/fpsyg.2024.1487355

**Published:** 2024-11-18

**Authors:** Qi Zhang, Heng Cao

**Affiliations:** Shandong Provincial Hospital Affiliated to Shandong First Medical University, Jinan, Shandong, China

**Keywords:** maintenance hemodialysis, dyadic coping, marital adjustment, post-traumatic growth, subject-object interdependence model

## Abstract

**Aim:**

To measure the relationships between dyadic coping, marital adjustment, and post-traumatic growth in patients with maintenance hemodialysis and their spouses.

**Background:**

Post-traumatic growth is common in patients facing maintenance hemodialysis. However, studies tend to focus on these patients as individuals rather than as part of a couple. Dyadic coping in a couple is important for their marital adjustment; however, little is known about how a couple's dyadic coping influences their marital adjustment and impacts their posttraumatic growth.

**Design:**

Cross-sectional study.

**Methods:**

A questionnaire survey was conducted among patients receiving maintenance hemodialysis and their spouses. Patients were recruited between December 2021 and October 2022 using convenience sampling from the blood purification centers of two first-class tertiary hospitals. A total of 230 couples (460 individuals) of patients receiving maintenance hemodialysis and their spouses were enrolled. A general condition questionnaire, the dyadic coping scale, and the Locke-Wallace marital adjustment scale were used for assessments and data entry and analysis were performed using EpiData 3.1, SPSS 25.0, and Mplus 8.4.

**Results:**

The dyadic coping of patients receiving maintenance hemodialysis was positively correlated with both marital adjustment and posttraumatic growth, as was the dyadic coping of their spouses. The marital adjustment of both patients and their spouses was found to partially mediate the association between dyadic coping and posttraumatic growth.

**Conclusion:**

Subject-mediated effects between the levels of dyadic coping, marital adjustment, and posttraumatic growth were established for both patients receiving maintenance hemodialysis and their spouses, and it was found that the marital adjustment in both showed varying degrees of mediation in the relationship between their dyadic coping and posttraumatic growth.

**Impact:**

The findings of the study suggest that attention should be given to promoting dyadic coping behaviors in patients receiving maintenance hemodialysis and their spouses. It is recommended that nurses offer both education and support to couples to promote dyadic coping.

## 1 Introduction

Maintenance hemodialysis (MHD) is the main form of replacement therapy for patients with end-stage renal disease (ESRD) resulting from chronic renal failure (Qiu et al., 2021), with 89.5% of patients with ESRD in China receiving MHD treatment. According to the latest census data released in the China Kidney Disease Network Data Report, ~402.18 patients per million receive MHD in China (Li and Liang, 2019; Wang et al., 2019). Although MHD treatment can prolong the survival of patients with ESRD, it is often associated with various issues, owing to the multimorbid nature of CKD, the requirement for multidisciplinary care, the high frequency of polypharmacy and the high rates of hospitalization and death, all of which affect the quality of life of patients (Bello et al., 2024; Chesnaye et al., 2024; Rehmann-Sutter, 2019). Furthermore, the treatment affects not only the physical and mental health of the patient but also their spouses who shoulder the main responsibility of care. Studies have found that the person most involved in patient care is the spouse (Hussien et al., 2021) and the burden of long-term care adversely affects their psychological balance (Rodakowski et al., 2012). Thus, MHD imposes stress on both the patient and their spouse and has a profound impact on the physical and mental health and marital satisfaction of couples.

Studies have shown that couples who view illness as a “couple experience” exhibit better personal and binary adaptability and greater relationship satisfaction than couples who view illness as a personal stressor (Stulz et al., 2022). In these situations, Bodenmann considered that couples depend on each other while coping with stress, helping each other during the process, and thus maintaining marital stability and promoting the mental health of both parties (Bodenmann, 2005; Ding and Mei, 2018). The concept of the systematic transaction model (STM) is dyadic coping, which refers to the shared reactions and strategies of couples to binary stress events (Bodenmann, 2005). These include positive dyadic coping behaviors, such as mutual support, joint coping, and communication when coping with disease-related stress, as well as negative dyadic coping styles, such as avoiding blame (An, 2020). The STM indicates that couples can reduce stress and strengthen marital adjustment through dyadic coping, thus maintaining and strengthening the marital bond. To date, research on dyadic coping both at home and abroad has focused on cancer (Badr et al., 2010; Shi, 2021), diabetes (Bai, 2021), stroke (Yang, 2021), and other fields, while research on chronic kidney disease is limited.

The Actor-Partner Interdependence Mode (APIM) proposed by Kenny and Ledermann (2010) represents a new method for data analysis, as it not only analyzes the relationship between the dependent variable in the research (such as the patient) and an independent variable, but can also analyze the relationship of the dependent variable with an independent variable associated with another part (such as the spouse) in the paired relationship, and can thus represent the overall relationship between the patient and spouse more scientifically and comprehensively (Sun et al., 2022).

Thus, the present study is based on the perspectives of both patients undergoing hemodialysis and their spouses, evaluating them as a whole. By analyzing the impact of the dyadic coping strategies of patients receiving maintenance hemodialysis and their spouses on marital adjustment, the findings provide a scientific reference for the identification of key targets and formulating intervention the formulation of effective interventional measures in clinical practice.

## 2 Backgroud

### 2.1 The relationship between dyadic coping and marital adjustment

The quality and degree of supportive dyadic coping between couples has an impact on affects both the degree strength of the marital intimacy bond and each other's mental health (Sun et al., 2022). Longitudinal studies on patients with breast cancer and their partners found that the higher the degree of breast cancer patients and partners dyadic coping, the better the marital adjustment (Rottmann et al., 2015). It has thus been suggested that there is a correlation between dyadic coping and marital adjustment. However, there are few studies on MHD patients and their spouses in China, and this topic thus requires further exploration to provide a quantitative basis for developing interventions to improve marital quality among MHD patients and their spouses.

### 2.2 The relationship of dyadic coping with post-traumatic growth

Active dyadic coping has been found to improve post-traumatic growth (PTG) levels in patients (Fujimoto and Okamura, 2021) and may thus be an effective strategy for enhancing PTG. In a previous study on the association between marriage quality and PTG in patients with cancer, we found that marriage quality was associated with greater positivity in dyadic relationships (Canavarro et al., 2015), while a study of 133 patients with breast cancer patients found that excessive intimacy between couples had a negative impact on PTG (Bodenmann and Randall, 2012). The mechanism by which marital adjustment affects PTG in MHD patients and spouses is unclear.

## 3 The study

### 3.1 Aim

The aim of this study was to evaluate the associations between dyadic coping in the spouses of patients undergoing hemodialysis, marital adjustment, and PTG.

### 3.2 Design

A cross-sectional study was conducted.

### 3.3 Participants

The study included patients with ESRD who were undergoing maintenance hemodialysis treatment and their spouses. The patients were recruited from the blood purification centers of two tertiary hospitals from December 2021 to October 2022, and were selected using convenience sampling. A cross-sectional survey method was used to conduct a questionnaire survey of the patients and their spouses. Patient inclusion criteria: (a) regular dialysis more than 1 month; (b) married and patient's primary caregiver is spouse; (c) stable condition, with no impairment of consciousness; (d) voluntary participation and the provision of written informed consent for study participation. Patient exclusion criteria: (a) being unable to understand the content of the questionnaire; (b) suffering from other serious physical diseases. Spouse inclusion criteria: (a) spouse of MHD patient included in the study; (b) literate and able to communicate normally; (c) voluntary participation and the provision of signed informed consent. Spouse exclusion criteria: (a) being unable to understand the content of the questionnaire; (b) suffering from serious physical conditions. Following Hoelter's (1983) guidelines, a sample size of at least 200 is recommended for path analysis to produce statistically reliable results. To meet this criterion 230 pairs of patients and spouses were initially recruited for the study, thus meeting the sample size requirements.

### 3.5 Procedure

Before the study, the investigator informed the supervisors of the sampled hospitals about the purpose and process of the study, and also obtained their approval. The collaborators selected by the researcher informed the nurses in the department about the study and instructed them to recruit participants.

### 3.6 Instruments

#### 3.6.1 General information

The general information questionnaire included information on gender, age, educational level, marriage age, monthly income, and other demographic information on MHD patients, as well as disease-related information such as the duration of dialysis treatment, primary disease type, and number of complications

#### 3.6.2 Dyadic coping inventory

The dyadic coping inventory (DCI) scale was developed by Bodenmann (2008) to evaluate the quality of communication and joint coping between both parties in a close relationship when facing pressure. Xu et al. (2016) examined the Chinese version of the DCI's factorial structure, measurement invariance (MI), and construct validity of test scores based on data from 474 Chinese couples. The scale contains 37 items and six dimensions, among which four dimensions, namely, stress communication, supportive coping, authorized coping, and negative coping are included for both individual perceived self-coping and individual perceived spousal coping. The joint coping dimension only covers the joint coping of both spouses as perceived by the patients, and two items in the dimension of coping quality evaluation were not included in the total score. Negative coping support was scored using a 5-point Likert scale, ranging from 1 (rarely) to 5 (very frequently). The total score was 35–175, with 111 as the critical value, 111–145 as the normal level, and scores above 145 as higher than the normal level. The higher the score, the greater the number of supportive coping behaviors and the better the dyadic coping.

#### 3.6.3 Locke-Wallace Marriage Adjustment Scale

The Locke-Wallace Marital Adjustment Test (LWMAT) was used to measure the quality and satisfaction of the relationships between MHD patients and their spouses. Wang and Wang (1999) translated this scale into Chinese version, and be used to Chinese couples. The scale has 15 items and four dimensions, including communication, sexual life inclusiveness, emotion, and differences in values. The total score is 0–158 points with higher scores indicating greater quality and satisfaction, and scores below 100 points representing marital disorder.

#### 3.6.4 Post-traumatic growth inventory

The Chinese version of the post-traumatic growth inventory (PTGI) was revised by Wang (2011) to measure the post-traumatic growth of MHD patients and their spouses. The scale has 20 items and five dimensions, and is scored on a 6-point Likert scale (0 = none, 5 = very much) with a total score of 0–100, with higher scores indicating higher levels of post-traumatic growth.

### 3.7 Data analysis

Data were analyzed using SPSS version 26.0 (IBM Corp, Armonk, NY, USA) and Mplus 8.4 software, with a significance level of α = 0.05. Normally distributed data are presented as mean ± standard deviation, while non-normally distributed data are shown as median and interquartile interval. Count data are presented as frequencies and percentages. Pearson correlation analysis was used for normally distributed data and Spearman rank correlation analysis for non-normally distributed data. To explore the subject-object effects of dyadic coping and marital adjustment on post-traumatic growth, a subject-object interaction-mediated model was constructed by Mplus. Mediating effects were evaluated by bootstrapping with a sample size of 5,000.

## 4 Results

### 4.1 Demographic characteristics

A total of 230 couples, including patients and their spouses, were enrolled in the study. Of these, 135 patients were male (58.7%) and 95 (41.3%) were female. Patient ages ranged from 28 to 82 years old, with an average age of 59.62 ± 10.59 years (Table 1).

**Table 1:** General demographic data of maintenance hemodialysis and spouses and disease data of patients.

**Variable**	**Patients (*****n*** = **230)**		**Spouses (*****n*** = **230)**	
	***N*** **(%)**		***N*** **(%)**	
**Gender**				
Male	145	63.04	85	36.96
Female	85	36.96	145	63.04
**Age**				
≤ 45	25	10.90	24	10.43
46–64	120	52.20	124	53.91
≥65	85	37.00	82	35.65
**Marriageable age**				
≤ 10	7	3.00	7	3.00
11–20	20	8.70	20	8.70
21–30	47	20.43	47	20.43
31–40	83	36.09	83	36.09
>40	73	37.74	73	37.74
**Education level**				
Junior high school and below	106	46.09	110	47.82
High school or technical secondary school	64	27.83	69	30.00
College degree or bachelor degree or above	60	26.08	51	22.17
**Type of employment**				
Employed	45	19.57	51	22.17
Unemployed	185	80.43	179	77.83
**Medical insurance**				
Medical insurance for urban workers	135	58.70		
Medical insurance for urban and rural residents	70	30.40		
Own expense or otherwise	25	10.90		
**Per capita monthly household income (RMB)**				
<1,000	48	20.87		
1,000–3,000	98	42.61		
3,000–5,000	58	25.22		
More than 5,000	26	11.30		
**Age of dialysis (month)**				
≤ 12	37	16.09		
13–60	111	48.26		
>60	56	24.35		
>120	26	11.30		
**Primary disease**				
Chronic glomerular nephritis	43	18.70		
Diabetic nephropathy	88	38.26		
Hypertension kidney disease	50	21.74		
**Comorbidity**				
None	11	4.78		
1	80	34.78		
2	88	38.26		
More than 3	51	22.17		

### 4.2 Comparison of dyadic coping, marital adjustment, and post-traumatic growth scores in hemodialysis patients

Paired-sample *t*-tests were used to analyze differences in dyadic coping, marital adjustment, and post-traumatic growth scores in MHD patients and their spouses. This showed that the mean total dyadic coping score of the patients was 116.87 ± 10.69 points, while that of the spouses was 116.04 ± 94.0 points. In terms of the perceived self-support coping, perceived self-authorization coping, perceived self-negative coping, and perceived spouse pressure communication dimensions, the patients scored significantly higher than their spouses (all *P* < 0.05). However, there were no significant differences in the total scores of dyadic coping and the dimensions of shared coping between patients and spouses and marital adjustment scores did not differ significantly between the hemodialysis patients and their spouses. The total scores for post-traumatic growth, personal strength, and new possible dimensions were lower in the patients relative to those of their spouses, with a significant difference in the post-traumatic growth scores (*P* < 0.05) (Table 2).

**Table 2:** Dyadic coping and marital adjustment scores in hemodialysis patients and their spouses.

	**Item**	**Patient ($\bar{x}\pm s$)**	**Spouse ($\bar{x}\pm s$)**	***t***	***P***
Total dyadic coping score	35	116.87 ± 10.69	116.04 ± 9.40	1.332	0.184
Self-perception of dyadic communication	4	13.08 ± 2.25	11.00 ± 2.26	10.502	<0.001
Self-supportive dyadic coping	5	15.97 ± 2.49	17.14 ± 2.04	−6.631	<0.001
Self-authorized coping, and	2	5.52 ± 1.37	7.40 ± 1.31	−14.146	<0.001
Self-negative dyadic coping	4	15.46 ± 1.99	16.17 ± 1.88	−4.485	<0.001
Perceived spouse dyadic communication	4	11.64 ± 2.17	12.93 ± 1.82	−7.534	<0.001
Perceived spouse supportive dyadic coping	5	16.94 ± 2.16	15.36 ± 2.53	8.768	<0.001
Perceived spouse delegated dyadic coping	2	7.24 ± 1.49	5.48 ± 1.44	12.044	<0.001
Perceived spouse negative dyadic coping	4	15.98 ± 2.13	15.50 ± 2.12	2.827	0.005
Common dyadic coping	5	15.03 ± 2.27	15.04 ± 2.31	−0.105	0.916
Marital adjustment	15	105.35 ± 14.839	106.52 ± 15.10	−1.288	0.199
Post-traumatic growth	20	53.62 ± 11.36	55.59 ± 9.07	−2.580	0.010

### 4.3 Correlations between dyadic coping, marital adjustment, and post-traumatic growth in hemodialysis patients

The Pearson correlation analysis showed that dyadic coping was positively correlated with both marital adjustment and post-traumatic growth (*P* < 0.01). The spouse dyadic coping scores were positively correlated with marital adjustment and post-traumatic growth (*P* < 0.01) (Table 3).

**Table 3:** Correlations between dyadic coping, marital adjustment, and post-traumatic growth in hemodialysis patients.

**Variable**	**Patient dyadic coping**	**Spouse dyadic coping**	**Patient marital adjustment**
Patient dyadic coping	–	–	–
Spouse dyadic coping	0.568^**^	–	–
Patient marital adjustment	0.633^**^	0.468^**^	–
Spouse marital adjustment	0.436^**^	0.584^**^	0.576^**^

^**^P < 0.05.

### 4.4 The mediating role of subject-object interdependence in dyadic coping, marital adjustment, and post-traumatic growth of maintenance hemodialysis patients and their spouses

**Figure 1:**
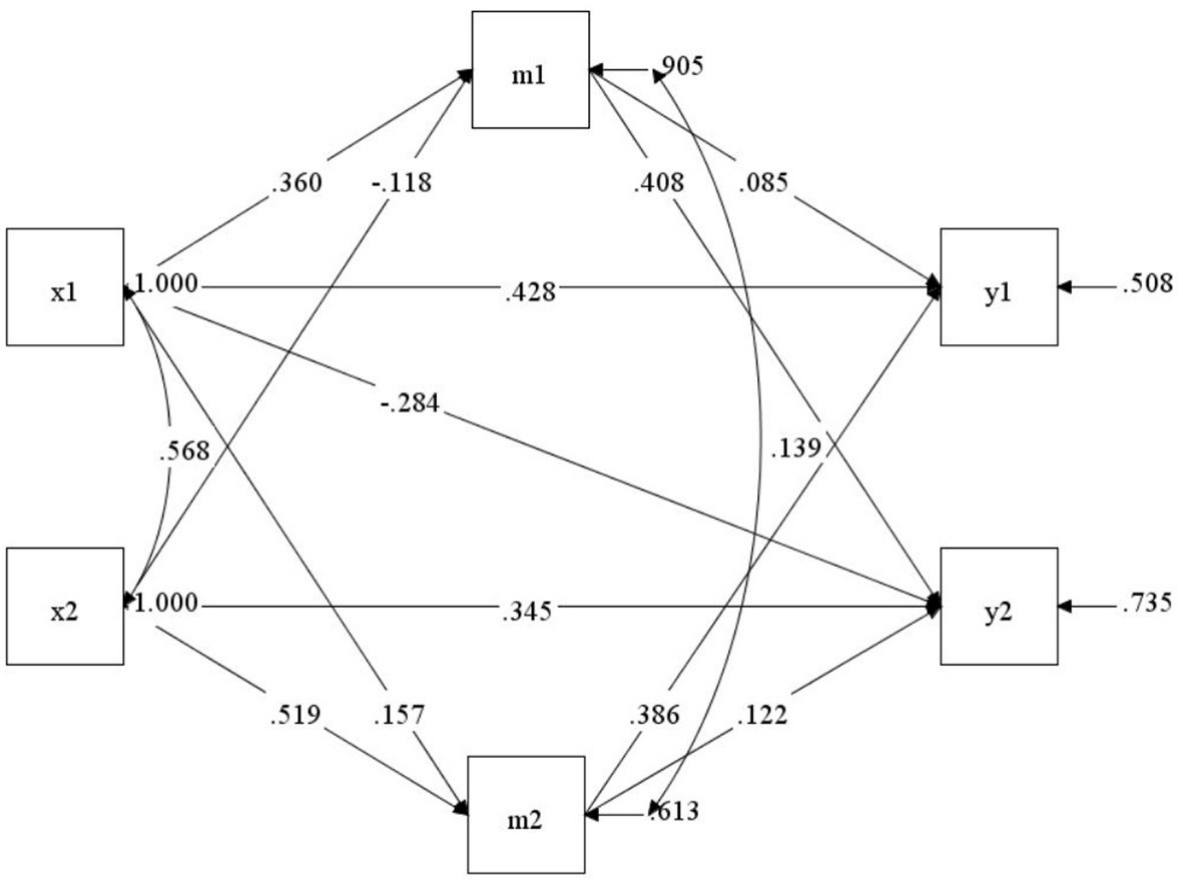
The mediating model of subject-object interdependence in marital adjustment between dyadic coping and post-traumatic growth. x1, m1, and y1, respectively, represent the patient's dyadic coping, marital adjustment, and post-traumatic growth; x2, m2, and y2 represent the spouse's dyadic coping, marital adjustment, and post-traumatic growth, respectively.

Bootstrap tests were performed on the mediating effect, and the results showed self-marital adjustment played a partial mediating role in the relationship between dyadic coping strategies of hemodialysis patients and their spouses and their post-traumatic growth (β = 0.031, 95% CI = 0.008–0.059 and β = 0.063, 95% CI = 0.011–0.122). This indirect effect between subjects was significant, demonstrating the subject-mediated effect of marital adjustment of the patients and their spouses in their own dyadic coping and post-traumatic growth relationship. At the same time, the marital adjustment of the other party played a partial mediating role (β = 0.061, 95% CI = 0.032–0.097 and β = −0.048, 95% CI = 0.088–0.013), demonstrating the object-mediated effect of the marital adjustment of the other party in their own dyadic coping and post-traumatic growth relationship. The mediating role of marital adjustment between patients and spouses in the relationship between the dyadic coping strategies of spouses and the post-traumatic growth of patients (β = −0.010, 95% CI = −0.026 to −0.002 and β = 0200, 95% CI = 0.155–0.255) indicated that the marital adjustment of patients and their spouses played a partial mediating role in the relationship between the patient's dyadic coping and spouse's post-traumatic growth (β = 0.147, 95% CI = 0.103–0.198 and β = 0.019, 95% CI = 0.004–0.044), establishing the object-mediation effect of patient and spouse marital adjustment in the relationship between the patient's dyadic coping and the spouse's post-traumatic growth. Details are provided in Table 4 and Figure 1.

**Table 4:** Bootstrap test and effect value of the subject-object interdependence mediating model between dyadic coping and post-traumatic growth.

**Effect**	**B**	**SE**	**95% CI**		***P***
**Patient dyadic coping**→**patient post-traumatic growth**				
Total effect	0.519	0.047	0.443	0.599	<0.001
Direct effect	0.428	0.046	0.352	0.506	<0.001
Total indirect effect	0.091	0.023	0.056	0.131	<0.001
Actor-actor simple IE (patient dyadic coping → patient marital adjustment → patient post-traumatic growth)	0.031	0.016	0.008	0.059	0.023
Partner-partner simple IE (patient dyadic coping → spouse marital adjustment → patient post-traumatic growth)	0.061	0.019	0.032	0.097	0.001
**Spouse dyadic coping**→**spouse post-traumatic growth**				
Total effect	0.360	0.058	0.263	0.454	<0.001
Direct effect	0.345	0.065	0.236	0.450	<0.001
Total indirect effect	0.015	0.040	−0.053	0.080	0.679
Actor-actor simple IE (spouse dyadic coping → spouse marital adjustment → spouse post-traumatic growth	0.063	0.033	0.011	0.122	0.020
Partner-partner simple IE (spouse dyadic coping → patient marital adjustment → spouse post-traumatic growth)	−0.048	0.023	−0.088	−0.013	0.034
**Patient dyadic coping**→**spouse post-traumatic growth**				
Total effect	−0.118	0.053	−0.208	−0.034	0.028
Direct effect	−0.284	0.053	−0.373	−0.198	<0.001
Total indirect effect	0.166	0.031	0.119	0.220	<0.001
Partner-partner simple IE (patient dyadic coping → patient marital adjustment → spouse post-traumatic growth)	0.147	0.030	0.103	0.198	<0.001
Partner-partner simple IE (patient dyadic coping → spouse marital adjustment → spouse post-traumatic growth)	0.019	0.012	0.004	0.044	0.049
**Spouse dyadic coping**→**patient traumatic growth**				
Total effect	0.157	0.045	0.084	0.231	<0.001
Direct effect	−0.033	0.047	−0.110	0.045	0.480
Total indirect effect	0.190	0.030	0.142	0.247	<0.001
Spouse dyadic coping → spouse marital adjustment → patient traumatic growth	0.200	0.030	0.155	0.255	<0.001
Spouse dyadic coping → patient marital adjustment → patient traumatic growth	−0.010	0.007	−0.026	−0.002	0.105

## 5 Discussion

### 5.1 Current status of dyadic coping levels in maintenance hemodialysis patients and their spouses

In this study, the mean dyadic coping score of hemodialysis patients was found to be 116.87 ± 10.69 points, and that of their spouses was 116.04 ± 9.40 points. According to the dyadic coping scoring standard, total scores between 111 and 145 are considered to be within the reference range. Therefore, the dyadic coping scores of both the patients and their spouses were within the normal range, and there was still room for improvement. The results of this study are similar to those of Cai et al. (2021) in patients with breast cancer but higher than those reported in a study by Ye et al. (2022) on 320 pairs of middle-aged and elderly patients with gynecological cancer and their spouses. The present study found no significant difference in the total dyadic coping score between patients and spouses, consistent with the findings of Cai et al. (2021). Perhaps because stress concerning disease spreads from the patient to the spouse in close relationships, stress perception is relatively consistent between both parties, with both being profoundly affected by the disease experience.

The negative dyadic coping scale in this study used a reverse-scoring method, that is, the higher the score of the negative dimension, the less the possibility of negative coping in life and the more positive the dyadic coping. In this study, both spouses were found to exhibit negative coping, with each perceiving the other's negative coping. However, the self-negative coping scores of the patients were lower than those of their spouses, while their perceived negative coping scores of the spouses were higher than those of their spouses. These differences were statistically significant, indicating that patients had higher levels of negative coping in daily life. The possible reason is that ESRD is an irreversible chronic progressive disease, which requires long-term dependence on dialysis to maintain life. Together with increasing the patient's physical discomfort, their economic status and interpersonal relationship are also seriously affected (Yang and Gao, 2022), making it easier for them to adopt negative coping styles.

### 5.2 Maintenance hemodialysis patients and spouses need to improve their marital adjustment levels

Marital adjustment refers to the mutual adjustment of couples within a specific time period that can enhance the individual's perception of spouse support and relationship satisfaction. The results of this study showed that the total marital adjustment scores of maintenance hemodialysis patients and their spouses were 105.35 ± 14.84 and 106.52 ± 15.10 points, respectively, which is similar to the results of a survey by Jieyu et al. (2022) on marital adjustment in couples undergoing first-time IVF but lower than the scores reported by Han and Xu (2021) on marital adjustment in infertile couples. It is possible that the reasons may be related to discomfort and distress caused by dialysis. Most patients tend to focus on their own pain, often ignoring the feelings of their spouses. The spouses represent the main caregivers for patients receiving long-term hemodialysis treatment, shouldering the responsibilities of taking care of the patient's daily living activities, accompanying them for dialysis treatment, supervising medication, and controlling their diet and water drinking. They can thus experience long-term physical and mental exhaustion, affecting emotional communication between husband and wife, leading to complex changes in their relationship, and potentially increasing the likelihood of conflict (Wawrziczny et al., 2021), which is not conducive to marital adjustment. In addition, female patients are often affected by reduced fertility or kidney disease, while male patients can be affected by reduced sexual function, potentially leading to feelings of loneliness, shame, or embarrassment. This can adversely affect the relationship, as when spouses see that their partner is experiencing physical pain and weakness, it is difficult to feel sexual attraction (Schembri Lia and Abela, 2020), thus reducing marital intimacy. Therefore, even if couples involved in hemodialysis have stable marital relationships, the quality of the relationship is likely to be reduced.

### 5.3 Low levels of post-traumatic growth in maintenance hemodialysis patients and their spouses

The results of this study showed that the total post-traumatic scores of hemodialysis patients and their spouses were 53.62 ± 11.36 and 55.59 ± 9.07, respectively, lower than those reported by Wang and Zhang (2021). Most maintenance hemodialysis patients and their spouses felt uncertain about the disease development and future life, and it was difficult to distinguish positive from painful emotions, indicating that the level of post-traumatic growth required improvement.

In this study, the results of the different dimensions of post-traumatic growth showed that the scores of both patients and their spouses in the personal strength dimension were higher, with the scores of the spouses in this dimension significantly higher than those of the patients (*P* < 0.01). The reasons were analyzed. After experiencing dialysis events, the families of patients make necessary changes in their lives, discovering their vulnerabilities and finding new internal strengths (Levkovich et al., 2023). Spouses often take over care tasks, help patients manage their disease, deal with the minutiae of daily life, and are open to accepting new things and ideas in the process. Spouses thus tend to experience greater growth in the face of adversity. In addition, the spouse is usually the main confidant of the patient, and thus becomes stronger with better-developed personal strength while guiding the patient (Bertschi et al., 2021). The dimension of life perception scored second, with the dimension of new possibility showing the lowest score. This may be due to the multiple comorbidities and obvious symptom burden (such as fatigue, insomnia, and itching) of the patient, which leads to the patient's family easily becoming immersed in the long-term painful experience associated with disease, potentially leading to a negative outlook and difficulties in discovering new possibilities in life. Medical professionals should thus encourage both patients and their spouses to actively explore new possibilities in life (He et al., 2024), cultivating and exploring new interests that will help divert attention from illness, making life more fulfilling, promoting a more positive outlook, and helping the recovery of their families.

### 5.4 Correlation between dyadic coping, marital adjustment, and post-traumatic growth in maintenance hemodialysis patients and their spouses

The dyadic coping style of hemodialysis patients and their spouses was positively correlated with their levels of marital adjustment and post-traumatic growth. This is consistent with previous studies (Li, 2021). Couples go through a phase of relationship readjustment as they try to balance stress through dyadic coping. Both partners experience pain associated with the disease and thus require the resources to cope with the consequences together, emphasizing the patient and partner roles, “you” and “me,” but also functioning as a whole. Intimate relationships can act as buffers against stress (Li et al., 2022; Zhou et al., 2024), and help couples focus on unity, strength, and resilience (Meuleman et al., 2024). Dyadic coping has two main functions, namely, stress-related functions and relationship-related functions. Stress-related functions are associated with stress reduction, which may primarily affect one partner, may spread to the other, or affect both partners simultaneously. Dyadic coping can mitigate the negative effects of stress and maintain and restore the overall health of both partners. A second, more important function of dyadic coping is to enhance trust and intimacy between couples and constructively resolve conflicts. Positive dyadic coping enhances a couple's ability to adapt to the disease, and when couples understand, unite, and support each other, they become dependable resources for each other, assisting in the active achievement of common goals and the creation of new emotional experiences. Helping each other during stressful times enhances the quality of their relationship. Previous studies have shown that sharing helps foster relationships (Zhaoyang et al., 2018), while reduced sharing introduces compromise in the relationship where couples do not discuss and resolve their concerns, avoiding discussion of the concerns which weakens the unity of the relationship (Brandao et al., 2017), leading to loneliness and persistent worry in coping with illness, and reduced intimacy. The dyadic coping of hemodialysis patients and their spouses was found to be significantly positively correlated with their post-traumatic growth. This could be due to one partner strengthening the sense of intimacy through supportive understanding, with both partners openly communicating their thoughts and feelings about the disease, thus reducing their sensitivity to stressful events and promoting a positive attitude toward hemodialysis.

### 5.5 Matrimonial adjustment mediates the interaction between dyadic coping and post-traumatic growth in maintenance hemodialysis patients and their spouses

The results of this study suggest that marital adjustment has a subject-object mediating effect between dyadic coping and post-traumatic growth in maintenance hemodialysis patients and their spouses. Specifically, marital adjustment partially mediates the relationship between dyadic coping and post-traumatic growth in hemodialysis patients and their spouses. This is consistent with the findings of Suo et al. (2021). The coping styles of dialysis patients and their spouses positively affect their marital adjustment, and marital adjustment also positively affects their post-traumatic growth. That is to say, the stronger the ability of both patients and spouses to cope with disease together, the better their marital adjustment, which positively affects the post-traumatic growth of both patients and spouses. Analysis of the reasons suggests that a greater frequency of positive coping behaviors, such as joint coping and stress communication, and a lower frequency of negative coping behaviors are more likely to benefit communication and understanding between the husband and wife in the daily management of the disease. Open communication allows both parties to understand each other's common expectations, leading to better adaptation to the disease, sharing and encouraging one another, and thus promoting the positive psychological experience of both spouses after coping with stressful events. The results are consistent with those of Sun et al. (2022) in cancer couples. The system interaction model proposed by Bodenmann and Randall (2012) was also verified. Both parties rely on each other when coping with stress through their perception and evaluation of stress, working together to support each other and cope with stress together, thus strengthening marital adjustment while reducing personal stress, and promoting mental health. The couple, as an interdependent system, contributes to personal wellbeing, improving the effectiveness of adjustment, creating a comfortable atmosphere of equal communication, positive expression of needs, mutual affirmation, and effective coping strategies. At the same time, good marital adjustment helps to reduce the distress of the patient. The life crisis faced by the patient is a catalyst for growth, triggering major changes in behavior, values, and priorities (Henson et al., 2021). The patient's family reacts to the threat posed by the disease as a catalyst for reshaping life. The patient and the spouse begin to critically examine themselves and the whole world when dealing with threatening events. This self-consciousness on the part of the affected individual allows successful coping with traumatic events, during which the perceptions of others and the meaning of the event can be reassessed and positively reshaped. In other words, deliberation enables the integration of traumatic events and the discovery of new meaning. Post-traumatic growth does not originate from the trauma itself but from constant struggle and effort. The more partners identify with their relationship, the more they think and talk about it from an “us” perspective, the more satisfied and positive they are likely to be with their relationship.

The dual coping strategies of hemodialysis patients and their spouses were found to have inconsistent effects on each other's post-traumatic growth. An object effect was observed between the patient's dual coping strategy and the spouse's post-traumatic growth, while the object effect between the spouse's dual coping strategy and the patient's post-traumatic growth was not established. The reason for this may be due to the active participation of the patient in coping with stress from both spouses, which reduces the physical and mental pressure on the spouse and assists with their psychological adjustment. However, while the spouse may have a high level of one-way dyadic coping, the patient is still immersed in distress. When the patient is not attuned to their spouse, only one party actively takes on the pressure and responds positively. This type of coping rarely leads to a couple's mutual growth, although it can promote personal growth (Badr et al., 2010). Previous studies have shown that if one spouse communicates actively but the other remains unaware, the couple often performs worse (Stulz et al., 2022), and the pain may increase. Effective nursing measures should be instituted to guide the expression of feelings and allow active coping with the pressure, assisting in overcoming difficulties. Therefore, future investigations should evaluate the dyadic coping strategies of maintenance hemodialysis patients and their spouses, guide patients and their spouses to adopt positive and effective joint coping strategies, promote communication between both parties, improve marital satisfaction, and promote individual growth.

There was an object-mediating effect between marital adjustment by one party and the post-traumatic growth of the other, that is, the marital adjustment of the spouse of the hemodialysis patient plays a partial mediating role between their joint response and their post-traumatic growth, while the marital adjustment level of the patient plays a partial mediating role between the spouse's contribution to the joint response and their post-traumatic growth. This result suggests that the dyadic coping of one party not only affects their own level of marital adjustment but also impacts the marital adjustment of their spouse, which in turn affects their own and their spouse's levels of post-traumatic growth. This once again confirms that hemodialysis events are a common source of stress for both spouses that require response as a couple rather than two individuals. Previous studies have shown that marital adjustment can not only predict one's own post-traumatic growth but also predict a spouse's post-traumatic growth (Zhaoyang et al., 2018). Both patients with chronic disease and their spouses can jointly adopt positive dyadic coping strategies to promote intimate marital relationships. For example, constructive communication between the two parties, through discussing pressure, emotional communication, and providing practical support, allows the members of the couple to convey positive ideas and hopes to each other, helping to strengthen individual marital adjustment, alleviate psychological pressure, reduce the emotional burden on the patient and their spouse, and promote individual psychological transformation toward a positive direction, thus assisting individuals in adapting to hemodialysis events. This suggests that medical staff should not only focus on hemodialysis patients in clinical work but should also evaluate both patient and spouse from a binary perspective, and develop binary interventional measures for both partners. For example, providing psychological education on the benefits of dyadic coping benefits not only the overall marital relationships but also the sexual relationship. Interventional strategies should focus on promoting common coping strategies, such as identifying problems, helping each other in ways that reduce stress, fostering intimacy, and encouraging couples to recognize and reduce the use of negative coping strategies, such as offering unwilling or unintentional support and not taking their partner's stress seriously (Weiss et al., 2019), thereby promoting marital adjustment and enhancing post-traumatic growth.

## 6 Limitations

The findings of this study have limitations. Firstly, the sample source is not extensive enough, and future research should incorporate data from multiple centers and larger samples. Secondly, due to the limitations of cross-sectional studies, this research can only reveal correlations between variables and cannot determine causality. Thirdly, the results of this study rely on patient self-reports, which often depend on the recall and reporting of investigators, potentially leading to recall bias.

## 7 Conclusion

Both maintenance hemodialysis patients and their spouses were found to have relatively low levels of dyadic coping, marital adjustment, and post-traumatic growth, thus requiring further improvement. The dyadic coping of maintenance hemodialysis patients was positively correlated with marital adjustment and post-traumatic growth, and dyadic coping in the spouses was also correlated with marital adjustment and post-traumatic growth. Subject-object mediation effects between dyadic coping, marital adjustment, and post-traumatic growth of both maintenance hemodialysis patients and their spouses were established. There were different degrees in the mediating effect of marital adjustment between patients and their spouses. The dyadic coping level of couples could affect their level of marital adjustment, subsequently affecting their post-traumatic growth. Couple-centered interventions aimed at cultivating positive dyadic coping and mitigating negative dyadic coping might be beneficial. Therefore, medical personnel should develop targeted interventional programs for hemodialysis patients and their spouses to improve the dyadic coping skills, marital adjustment, and post-traumatic growth of couples (Feng et al., 2024; Han and Gao, 2024).

## Data Availability

The original contributions presented in the study are included in the article, further inquiries can be directed to the corresponding author.
